# Melatonin as a Therapeutic Agent for the Inhibition of Hypoxia-Induced Tumor Progression: A Description of Possible Mechanisms Involved

**DOI:** 10.3390/ijms221910874

**Published:** 2021-10-08

**Authors:** Sepideh Bastani, Moloud Akbarzadeh, Yeganeh Rastgar Rezaei, Ali Farzane, Mohammad Nouri, Mahsa Mollapour Sisakht, Amir Fattahi, Maryam Akbarzadeh, Russel J. Reiter

**Affiliations:** 1 Research Center for Pharmaceutical Nanotechnology (RCPN), Tabriz University of Medical Sciences, Tabriz 51368, Iran; sepidehbastani.88@gmail.com; 2 Stem Cell And Regenerative Medicine Institute (SCARM), Tabriz University of Medical Sciences, Tabriz 51368, Iran; moloodakbarzadeh@gmail.com; 3 Department of Cellular and Molecular Biology, Faculty of Biological Science, Azarbaijan Shahid Madani University, Tabriz 51368, Iran; 4 Department of Medical Biotechnology, Faculty of Advanced Medical Sciences, Tabriz University of Medical Sciences, Tabriz 51368, Iran; yeganerastgar@gmail.com; 5 Department of Health Information Management, School of Allied Medical Science, Tehran University of Medical Sciences, Tehran 11369, Iran; a-farzane@razi.tums.ac.ir; 6 Department of Reproductive Biology, Faculty of Advanced Medical Sciences, Tabriz University of Medical Sciences, Tabriz 51368, Iran; nourimd@yahoo.com; 7 Stem Cell and Regenerative Medicine Center of Excellence, Tehran University of Medical Sciences, Tehran 11369, Iran; m.molapoursisakht@erasmusmc.nl; 8 Department of Biochemistry, Erasmus University Medical Center, P.O. Box 2040, 3000 CA Rotterdam, The Netherlands; 9 Department of Obstetrics and Gynecology, Erlangen University Hospital, Friedrich-Alexander University of Erlangen–Nürnberg, Comprehensive Cancer Center ER-EMN, 91054 Erlangen, Germany; 10 Department of Cell Systems and Anatomy, UT Health, Long School of Medicine, San Antonio, TX 78229, USA; reiter@uthscsa.edu

**Keywords:** melatonin, cancer, antioxidant, apoptosis, angiogenesis, metastasis

## Abstract

Hypoxia has an important role in tumor progression via the up-regulation of growth factors and cellular adaptation genes. These changes promote cell survival, proliferation, invasion, metastasis, angiogenesis, and energy metabolism in favor of cancer development. Hypoxia also plays a central role in determining the resistance of tumors to chemotherapy. Hypoxia of the tumor microenvironment provides an opportunity to develop new therapeutic strategies that may selectively induce apoptosis of the hypoxic cancer cells. Melatonin is well known for its role in the regulation of circadian rhythms and seasonal reproduction. Numerous studies have also documented the anti-cancer properties of melatonin, including anti-proliferation, anti-angiogenesis, and apoptosis promotion. In this paper, we hypothesized that melatonin exerts anti-cancer effects by inhibiting hypoxia-induced pathways. Considering this action, co-administration of melatonin in combination with other therapeutic medications might increase the effectiveness of anti-cancer drugs. In this review, we discussed the possible signaling pathways by which melatonin inhibits hypoxia-induced cancer cell survival, invasion, migration, and metabolism, as well as tumor angiogenesis.

## 1. Introduction

Cancer is a major cause of morbidity and mortality worldwide [1]. Although genetic mutations have a decisive role in cancer development, many cancers are a consequence of environmental risk factors such as diet, smoking, pollutants, stress, inflammation, etc. [2]. Several features of cancer cells pave the way for tumor development, including persistent proliferation and insensitivity to growth suppressors, constant DNA replication, evasion of both apoptosis and immune surveillance, impaired energy metabolism, sustained angiogenesis, invasion, and metastasis [3]. 

Metastasis is the most common event that makes the treatment of cancer challenging. During tumorigenesis, some cancer cells readily undergo metastasis; this process begins with the dissociation of the cell’s tumor mass, and the invasion into the tumor microenvironment [3]. These invasive cells pass across the endothelial wall and enter into the blood and/or lymphatic circulatory systems, a process known as intravasation. Some of these circulating cells may escape the circulation (extravasation) and initiate growth at a distant site to produce subsets of the original tumor. If this new colony continues the proliferation it can form a secondary metastatic tumor [4]. In some cases, continued chemotherapy leads to treatment resistance. Chemoresistance occurs often with recurrent cancers. The recurrence of cancer is a result of surviving cancer stem cells; these cells play a central role in tumor regrowth [5].

Neoangiogenesis is a notable feature of tumors in which new vessels sprout from pre-existing blood vessel networks to provide vital nutrients and oxygen for cancer cell growth and proliferation [3]. It has been shown that the disruption of pro-angiogenic and anti-angiogenic regulators could lead to uncontrolled angiogenesis [5]. 

Hypoxia (oxygen tension less than 7 mmHg), which is sensed by hypoxia-inducible factors (HIFs), induces overexpression of the growth factors and cellular adaptation genes which subsequently promote angiogenesis, cancer cell survival, proliferation, and energy metabolism [6]. The newly created vessels are immature and leaky, and therefore the oxygenation and drug delivery are sometimes diminished in these vessels; accordingly, hypoxic tumors are usually resistant to chemotherapy. The hypoxic state in the tumor microenvironment may provide new therapeutic approaches to selectively destroy the hypoxic cells. In this regard, two distinct approaches have been proposed, including “bioreductive prodrugs” and “molecular target inhibitors” [7]. Moreover, targeting the pro-angiogenic factors or their receptors is considered a valuable strategy for limiting the growth and metastasis of tumors [8]. 

Melatonin (N-acetyl-5-methoxytryptamine), a multifunctional molecule, is produced in and released from the pineal gland and likely synthesized in the mitochondria of all other cells, where it is used locally and not released into the blood [9]. Many functions have been reported for melatonin, including the regulation of circadian rhythms and annual cycles of reproduction, antioxidant actions, and immune system regulation [10,11]. Additionally, melatonin has multiple anti-cancer properties such as anti-proliferation, anti-angiogenesis, immune system modulation, and apoptotic activities [12,13,14,15]. More interestingly, studies have demonstrated that melatonin modulated hypoxia-induced tumorigenesis [16,17,18], and co-administration of melatonin in combination with other therapeutic compounds increased the effectiveness of those treatments [19,20,21]. This review aims to describe the pathways involved in hypoxia-induced cancer development and more importantly explain how melatonin can possibly inhibit hypoxia-mediated tumor progression. Moreover, the current study provides possible mechanisms involved in the inhibition of hypoxia-induced tumor progression by melatonin. 

## 2. Hypoxia and Cancer (Tumor) Progression

Hypoxia occurs in many solid tumors and plays a role as a selective agent throughout metastatic transformation and progression [22]. Although hypoxia negatively affects tumor proliferation in some conditions, it mainly allows tumor cells to adapt to insufficient oxygen and nutrients and consequently enhances the activity and aggressiveness of cancer cells. Moreover, genomic changes occurring in the tumor cells under low oxygen conditions can make it feasible for them to survive. In turn, the excessive proliferation of cancer cells exaggerates the hypoxic state. As a result, a vicious circle of hypoxia and tumor progression develops [23]. Hypoxia is also associated with genomic instability and induces malignant phenotypes such as apoptosis resistance [24]. Furthermore, poor vascularity reduces tumor cell exposure to drugs during chemotherapy and oxidative damage during radiotherapy; thus, it is common for tumors to develop resistance to chemotherapy and radiotherapy under hypoxic conditions [23]. 

**Figure 1:**
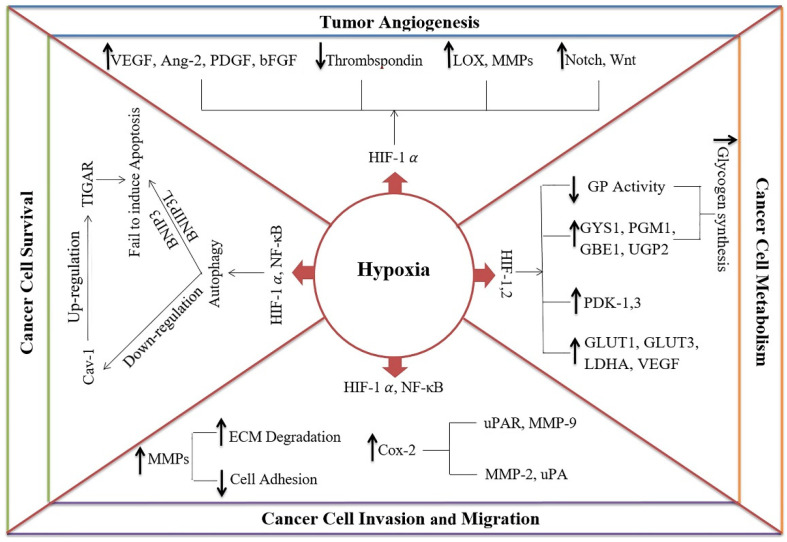
The mechanisms of hypoxia in cancer progression. Hypoxia enhances cancer cell survival by inducing autophagy via hypoxia-inducible factor-1 alpha (HIF-1α) and nuclear factor kappa-light-chain-enhancer of activated B cells (NF-κB). Subsequently, it causes (1) the down-regulation of caveolin-1 (Cav-1), leading to the up-regulation of TP53-inducible glycolysis and apoptosis regulator (TIGAR) and protects cells against oxidative stress and apoptosis, and (2) protection of the cells from apoptosis via BCL2 interacting protein 3 (BNIP3) and BCL2 interacting protein 3-like (BNIP3L). Hypoxia can induce tumor angiogenesis by (1) increasing proangiogenic factors such as vascular endothelial growth factor (VEGF), angiopoietin-2 (Ang-2), platelet-derived growth factor (PDGF), and basic fibroblast growth factor (bFGF), (2) decreasing angiogenesis inhibitors such as thrombospondin, (3) up-regulation of extracellular matrix (ECM) proteins, such as lysyl oxidase (LOX) and matrix metalloproteinases (MMPs), and (4) activating Notch and Wnt signaling pathways. Hypoxia increases cancer cell invasion and migration by the down-regulation of cell adhesion molecules and the up-regulation of ECM degradation molecules such as MMP-9 and urokinase-type plasminogen activator receptor (uPAR). Hypoxia affects the metabolic pathways to provide high energy for cancer cells by (1) enhancing the transcription of glucose transporters genes (GLUT1 and GLUT3), VEGF, and glycolytic enzymes (e.g., lactate dehydrogenase, LDHA), (2) inducing the expression of glycogenesis enzymes including phosphoglucomutase-1 (PGM1), glycogen synthase-1 (GYS1), UDP-glucose pyrophosphorylase 2 (UGP2) and 1,4-alpha-glucan branching enzyme 1 (GBE1), (3) reducing glycogen phosphorylase (GP) activity, and (4) diverting pyruvate from the citric acid cycle into lactate by pyruvate dehydrogenase kinases-1 and -3 (PDK-1 and -3).

Hypoxia up-regulates growth factors and cellular adaptation genes by increasing the levels of HIF proteins which have a significant impact on cancer progression [6]. Interestingly, mutations that cause either oncogene activation or tumor suppressor inactivation can increase HIF-1α expression in cancer cells [25]. Hypoxia positively induces survival, invasion, migration, metabolism, and angiogenesis in cancer cells, as discussed below (summarized in Figure 1).

### 2.1. Hypoxia Induces Cancer Cell Survival

The oxygen state determines whether a cell will or will not undergo apoptosis [26]. Moreover, based on the duration of exposure to hypoxia, the response of cancer cells can vary from death to survival. The cycling hypoxia-induced high production of reactive oxygen species (ROS) is associated with tumor cell survival and progression [27]. However, there are sometimes atypical actions regarding the role of the hypoxia-induced HIF pathway in cancer cell survival. For example, HIF-1 can either prevent cell death or induce apoptosis [28]. It is also reported that HIF-1 regulates insulin-like growth factor 2 (IGF-2), a crucial survival factor, in hypoxic tumor cells [29]. Hypoxia-related pathways including PI3K/AKT/mTOR, ERK, and the NF-ĸB are also involved in cancer cell proliferation and survival [30]. Hypoxia can lead to autophagy via HIF-1α and NF-κB. It is well-established that autophagy is a pro-survival process that generates nutrients and biomolecules required by rapidly growing cells, and this also protects the cells from apoptosis via Bcl-2 subfamilies such as BNIP3 (Bcl-2/adenovirus E1B 19 kDa interacting protein 3) and BNIP3L (Bcl-2/adenovirus E1B 19 kDa interacting protein 3-like) [31]. Hypoxia can also down-regulate caveolin-1 (Cav-1), and studies have demonstrated that loss of Cav-1 up-regulates TIGAR (TP53-induced glycolysis and apoptosis regulator) which protects cells against oxidative stress and apoptosis [31]. In summary, it can be postulated that hypoxia, at least in the short term, induces cancer cell survival by activating autophagy, suppressing apoptosis, and inducing metabolic adaptation [32].

### 2.2. Hypoxia Induces Tumor Angiogenesis 

One of the most significant effects of hypoxia is the induction of neoangiogenesis in the tumor [33]. Angiogenesis is a critical step in cancer progression that provides nutrients and oxygen [34]. For this purpose, the tumor forms a prerequisite vascular network not only by recruiting the host vessels, but also by forming new microvessels. The newly formed vasculature displays various irregularities in structure and function which result in abnormal blood flow and inefficient oxygen delivery to the tumor cells, and consequently, the development of the hypoxic status [23]. Additionally, the enlargement rate of the tumor exceeds the growth of new blood vessels which also causes a relative hypoxic area, especially near the center of the tumor [35]. In a growing tumor, oxygen demand is increased but its availability decreased, which may help the hypoxia–angiogenesis cycle. Hypoxia induces a cascade of proangiogenic factors, including VEGF, angiopoietin 2 (Ang-2), platelet-derived growth factor (PDGF), and basic fibroblast growth factor (bFGF), while also reducing angiogenic inhibitors such as thrombospondin through HIF-1 [36]. There is some evidence that HIF-2α plays a role in the up-regulation of VEGF and its receptor [37]. 

VEGF and Ang-2 are the most prominent regulators of angiogenesis which are induced by hypoxia [38]. In this regard, Olaso et al. [39] have demonstrated that hepatic stellate cells existing in hypoxic conditions release VEGF during the formation of micrometastases. The development of macrometastases can be possible after the endothelial cells accumulate and form a sustainable stable vasculature. Moreover, hypoxia up-regulates extracellular matrix (ECM) proteins such as lysyl oxidase and matrix metalloproteinases (MMPs), which have a role in angiogenesis [40]. The MMP-inducers such as ECM metalloproteinase inducer (EMMPRIN/CD147) promote angiogenesis not only by acting as a protease, but also by increasing levels of the soluble VEGF isoforms [41]. Furthermore, membrane-type 1 matrix metalloproteinase (MT-MMP) is present in some cancer cells, and has a central role in the release of Sema4D, a tumor-inducing angiogenesis factor under hypoxic conditions [42]. Additionally, hypoxia down-regulates the soluble receptor of VEGF (known as sFlt-1, a VEGF antagonist), and thus increases VEGF activity [43,44]. Hypoxia-induced HIF-1α can also up-regulate the Notch signaling pathway which, along with Wnt signaling, determines the vascular density [45]. Finally, hypoxia promotes angiogenesis by stimulating proangiogenic factor IL-8 via activation of NF-κB [46]. The above-mentioned findings clearly show the role of hypoxia in tumor angiogenesis. However, further studies are required to define the underlying mechanisms and mediators that are involved in these processes. 

### 2.3. Hypoxia Induces Invasion and Migration of Cancer Cells

The first step in metastasis is the invasion of cancer cells between endothelial cells that allow them to enter the lymphatic or cardiovascular system for further spreading. Generally, cancer cell invasion begins with the degradation of the extracellular matrix by MMPs and the destruction of integrin adhesion [47]. The potential of cancer cells to alter extracellular matrix remodeling and digestion of the basement membrane also contributes to tumor progression and invasion [38,47]. Hypoxia leads to the detachment of tumor cells by down-regulating cell adhesion molecules, and by up-regulating the molecules involved in the degradation of integrin and cell attachment components such as MMP-9 and urokinase-type plasminogen activator receptor (uPAR) [48,49]. Hypoxia, by stabilizing microtubules and facilitating integrin localization in the cell membrane, also stimulates the cell motility which is needed for invasion and migration [47]. Hypoxia-induced NF-κB also up-regulates cyclooxygenase-2 (COX-2) and consequently the expression of some essential cell surface and cytoskeletal proteins required for tumor invasion, including matrix metalloproteinase-2 (MMP-2) and urokinase-type plasminogen activator (uPA) [23,50]. Moreover, the Rho family member A (RhoA) which is required for the activation of MT1-MMP is increased in the hypoxic microenvironment [47]. Furthermore, hypoxic macrophages can indirectly stimulate the secretion of MMPs [51]. 

### 2.4. Hypoxia Regulates the Metabolism of Cancer Cells

The ATP source in normal cells is mitochondrial oxidative phosphorylation, whereas in tumor cells it is cytosolic glycolysis in both normoxic (Warburg effect) or hypoxic (Pasteur effect) conditions [23]. The tumor cells change their glycolytic pathway to reduce oxygen consumption by increasing the rate of glucose uptake and lactic acid fermentation [52]. It has been reported that there is a correlation between lactate production and the metastatic spread of tumors [53,54]. This glycolytic processing is likely regulated by the hypoxic inducible factor (HIF-1) to increase transcription of genes encoding glucose transporters (GLUT1 and GLUT3), VEGF, and glycolytic enzymes (lactate dehydrogenase A, LDHA) [55]. For example, LDHA, a target of HIF-1, catalyzes the conversion of pyruvate to lactate which is crucial for tumor initiation, maintenance, and progression [56]. Moreover, HIF-1 increases pyruvate dehydrogenase kinases (PDK) 1 and 3 which reduce mitochondrial uptake of pyruvate and divert it for conversion into lactate by LDH [57]. Hypoxia can also increase glycogen synthesis as a survival strategy under harsh conditions; this process is carried out by HIF-1 and HIF-2 via up-regulation of glycogenesis enzymes, including phosphoglucomutase 1 (PGM1), glycogen synthase 1 (GYS1), glucose-1-phosphate uridylyltransferase (UTP), and 1,4-α glucan branching (GBE1) [52]. These collective data show that hypoxia induces several metabolic changes in favor of providing high energy for cancer cells.

## 3. Melatonin as an Inhibitor of Hypoxia-Induced Pathways

### 3.1. Melatonin Definition and Physiological Roles

Melatonin (N-acetyl-5-methoxytryptamine) has attracted a great deal of attention in various medical contexts. Although this molecule is produced and secreted by the pineal gland, especially at night, all cells likely produce melatonin where it is used locally [58,59]. In vertebrate cells, melatonin synthesis happens in mitochondria, which contain much higher concentrations of this molecule relative to other organelles. Importantly, these high levels of melatonin are maintained even after pinealectomy [60]. Mitochondria as a source of melatonin are also supported by the observation that isolated mitochondria from oocytes could synthesize melatonin [61]. 

The roles of this molecule in the regulation of the sleep–wake cycle, circadian and circannual rhythms, seasonal adaptations, reproduction, and immune response have been well documented. Over the last four decades, numerous reports have confirmed that melatonin acts as an endogenous oncostatic agent for many cancer types [59,62,63,64]. The anti-cancer effects of melatonin are often mediated by both receptor-dependent and independent mechanisms [65,66]. The receptor-dependent mechanisms involve the G-protein receptor-related family of melatonin receptors, MT1 (Mel1a) and MT2 (Mel1b), which inhibit the MAPK and PI3K signaling pathways. The receptor-independent mechanisms are mediated via direct inhibition of calmodulin and cAMP-related pathways by melatonin [67,68], and are related to its ability to modulate oxidative homeostasis [69]. Melatonin acts as an anti-tumor factor by interfering with different properties of cancer cells such as growth, proliferation, metastasis, angiogenesis, immune evasion, and cellular metabolism [70]. Many of these data have been elegantly summarized by Hill and colleagues [70]. 

### 3.2. Melatonin as a Proposed Therapeutic Factor for the Inhibition of Hypoxia-Induced Tumor Progression 

**Figure 2:**
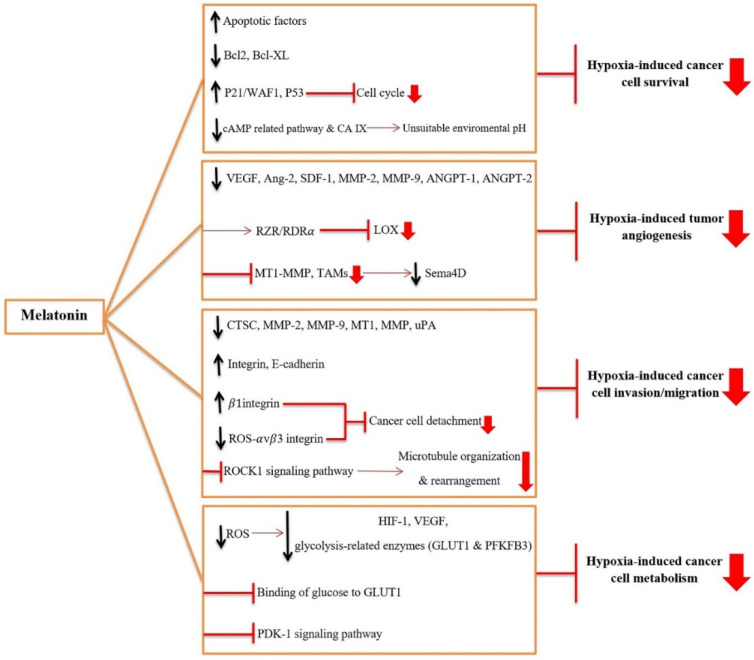
The mechanisms through which melatonin inhibits hypoxia-induced tumor progression. Melatonin inhibits the survival of hypoxic cancer cells by (1) up-regulating and activating the apoptotic factors, (2) down-regulating and inactivating anti-apoptotic factors [B-cell lymphoma 2 (Bcl-2) and B-cell lymphoma-extra large (Bcl-xL)], (3) blocking the cell cycle by up-regulating p21/WAF1 and p53, and (4) inhibiting carbonic anhydrase IX (CA IX) expression and activity and cAMP-related pathways to make an unsuitable environmental pH. Melatonin inhibits hypoxia-induced angiogenesis by (1) suppressing the activity of vascular endothelial growth factor (VEGF), angiopoietin-2 (Ang-2), stromal-derived factor 1 (SDF-1), matrix metalloproteinase-2 and -9 (MMP-2 and -9), angiopoietin-1 and -2 (ANGPT-1 and -2), (2) inhibiting the expression of lipoxygenase (LOX) via interacting with RZR/RORα nuclear receptor, and (3) blocking the hypoxia-induced tumor-associated macrophages (TAMs) and membrane-type 1 matrix metalloproteinase (MT1-MMP) activity and subsequently reducing Semaphorin-4D (Sema4D). Melatonin inhibits the hypoxia-induced invasion and migration of cancer cells by (1) decreasing levels of proteases including Cathepsin C (CTSC), MMP-2, MMP-9, MT1-MMP, and urokinase-type plasminogen activator (uPA), (2) up-regulating the adhesion proteins, such as integrin and E-cadherin, (3) suppressing oxidative-stress-induced detachment of cancer cells via overexpression of the β1 integrin and down-regulation of ROS-αvβ3 integrin-FAK/Pyk2 (focal adhesion kinase/proline-rich tyrosine kinase 2) signaling pathway, and (4) blocking hypoxia-induced microtubule organization and rearrangement via blocking the Rho-kinase 1 (ROCK1) signaling pathway. Melatonin disturbs hypoxia-induced cancer cell metabolism by (1) reducing reactive oxygen species (ROS) and down-regulating hypoxia-inducible factor-1 (HIF-1), VEGF and glycolysis-related enzymes such as glucose transporter 1 (GLUT1) and progestins activate 6-phosphofructo-2-kinase/fructose-2,6-bisphosphatase 3 (PFKFB3), and (2) competing with glucose in binding to GLUT1, and 3) inhibition of 3-phosphoinositide-dependent protein kinase 1 (PDK-1) signaling pathway.

As discussed in previous sections, hypoxia is an important factor in tumor progression that positively affects survival, angiogenesis, invasion, migration, and the metabolic status of cancer cells (see Section 2. Hypoxia and cancer (tumor) progression). Hypoxia also contributes to the radioresistance and chemoresistance of the tumor. Melatonin is a potent anti-tumor agent that likely inhibits various hypoxia-induced signaling pathways in cancer cells. Thus, we propose that a way by which melatonin inhibits cancer growth and progression and also improves therapeutic efficacy is the inhibition of hypoxia-induced survival, angiogenesis, migration, and invasion (Figure 2). The following section describes how melatonin prevents hypoxia-induced properties of cancer cells. 

#### 3.2.1. Melatonin Inhibits the Hypoxia-Induced Survival of Cancer Cells

Accumulating evidence has confirmed that hypoxia down-regulates apoptotic elements, including caspase-3, -8 and -9, cytochrome complex (Cyt c), Fas/FasL, and Bax in cancer cells and therefore supports these cell’s survival [14,71]. On the contrary, melatonin inhibits the survival of cancer cells by up-regulating/activating apoptotic components. Furthermore, melatonin down-regulates/inactivates Bcl-2 and Bcl-xL in hypoxic cancer cells [72,73]. Melatonin also blocks the cell cycle and up-regulates p21/WAF1 and p53, which subsequently inhibit the proliferation of hypoxic tumor cells [74]. Melatonin also decreases the expression of cyclin A and cyclin D in hypoxic cells, thereby regulating the cell cycle. Moreover, it has been shown that melatonin could reduce the proliferation of hypoxic pancreatic stellate cells [14].

Different hypoxia-induced signaling pathways may be targets for melatonin to inhibit cancer cell survival. For instance, hypoxia stimulates the adenylyl cyclase (AC)/cAMP/protein kinase A (PKA) signaling pathway to provide suitable microenvironmental pH for cancer cell survival [75]. In addition, hypoxia mediates overexpression of the carbonic anhydrase IX (CA IX) gene in an HIF-1α-dependent manner which acts as a pH regulator in the tumor [76]. Conversely, melatonin modulates cAMP-related pathways as well as CA IX expression and activity, and thereby makes the condition less suitable for cancer cells [77]. Moreover, it has been demonstrated that melatonin could increase and decrease the phosphorylation of respectively p38 and JNK in pancreatic stellate cells in hypoxic conditions, leading to a decrease proliferation of the cells [78]. Melatonin has been found to induce apoptosis by sensitizing the hepatocellular carcinoma cells to sorafenib and modulating autophagy through the PERK-ATF4-Beclin1 signaling pathway [79]. Another study showed that melatonin inhibited the proliferation of gastric cancer cells via the IRE/JNK/Beclin1 signaling pathway [80].

Hypoxia-related factors (e.g., HIF-1) up-regulate different cell survival factors such as transforming growth factor α (TGFα), endothelin 1 (EDN1), IGF-2, VEGF, and EPO [81], whereas melatonin suppresses these factors. For example, melatonin at a physiologic concentration (1 nM) down-regulates NF-κB, TGF, VEGF, and c-Myc and up-regulates p53 and p21 in breast cancer cells [74]. Moreover, in a study by Leon et al. [82], inactivation of NF-κB and PKC and down-regulation of EDN1 were observed in colon cancer cells following melatonin treatment. Moreover, the hypoxic condition triggerred various signaling pathways such as PI3K/AKT/mTOR, ERK, and NF-κB which are involved in cancer cell survival [30,83]. Melatonin at extremely high pharmacological doses (1 mM) reportedly inhibited these signaling pathways, possibly due to its toxic effect at this concentration [84]. 

One of the survival strategies against chemotherapy in hypoxic cancer cells is HIF-1-induced chemoresistance. The main player in this process is truncated VDAC1-ΔC (voltage-dependent anion channel 1) which acts as a channel to maintain ATP and inhibit apoptosis [85]. Kristinina et al. [86] revealed that co-treatment of melatonin and retinoic acid down-regulated VDAC1 and the activity of the electron transport chain complexes in HL-60 cells; therefore, it can be postulated that melatonin also puts the survival of chemoresistant cancer cells in danger. 

#### 3.2.2. Melatonin Inhibits the Hypoxia-Induced Angiogenesis of Tumors

The positive effect of HIF-1 on the expression of several proangiogenic factors such as VEGF, stromal-derived factor 1 (SDF-1), Ang-2, PDGF, bFGF, and angiopoietin (ANGPT) -1, -2, is well documented [36,81]. Melatonin exerts its anti-angiogenesis role mainly by reducing the levels of HIF-1 [87]. The augmented level of ROS in hypoxic cells leads to the inactivation of PHD and the stability of HIF-1α [73]. Melatonin suppresses the hypoxia-induced production of ROS and so reduces the stability of HIF-1α [18]. Furthermore, melatonin inhibits angiogenesis by suppressing the activity of VEGF, Ang-2, SDF-1, MMP-2, MMP-9, ANGPT-1, and ANGPT-2 [88,89]. Moreover, the inhibitory effect of melatonin on PDGF has been reported in liver fibrosis [90]; therefore, it can be assumed that melatonin may also attenuate PDGF levels in hypoxic cancer cells. 

Hypoxia increases ECM proteins such as LOX which is associated with angiogenesis [40,91], and, on the contrary, melatonin suppresses LOX expression via interacting with the RZR/RORα nuclear receptor [92]. Moreover, melatonin suppresses the production of Sema4D, an important angiogenic factor released by MT1-MMP and TAMs, by blocking the hypoxia-induced TAM activity [93]. It can be concluded that melatonin, directly and indirectly, inhibits hypoxia-induced angiogenesis in tumors by modulating HIF-1-induced angiogenic factors and HIF-1 levels/activity. 

#### 3.2.3. Melatonin Inhibits the Hypoxia-Induced Invasion and Migration of Cancer Cells

Hypoxia helps cancer cells to invade and migrate to other parts of the body. In fact, the hypoxic condition makes the invasion and migration of cancer cells possible by both the down-regulation of cell adhesion molecules and the up-regulation of proteases [48]. Hypoxia-induced HIF-1 mediates the up-regulation of ECM degradation enzymes (e.g. CTSC, MMP-2, MMP-9, MT1-MMP, uPA [81]. On the other hand, melatonin inhibits the migration and invasion of cancer cells by decreasing levels of several proteases including CTSC, MMP-2, MMP-9, MT1-MMP, and uPA [94]. Furthermore, melatonin has the potential to inhibit cancer cell migration via up-regulating the adhesion proteins, such as integrin and E-cadherin [74]. Melatonin also suppresses oxidative-stress-induced detachment of cancer cells via overexpressing the β1 integrin and down-regulation of ROS-αvβ3 integrin-FAK/Pyk2 signaling pathway [95,96]. HIF-1α overexpresses RhoA and Rho kinase 1 (ROCK1) leading to actin–myosin contraction and cell motility [17]. Moreover, Rho triggers the focal adhesion kinase (FAK) signaling pathway and consequently induces motility and an invasive phenotype of hypoxic cancer cells [97]. Interestingly, melatonin blocks hypoxia-induced microtubule organization and rearranges the microtubules via the ROCK1 signaling pathway [66,98]. Moreover, Doganlar et al. [99] showed that melatonin could suppress the invasion of human glioblastoma tumor spheroids by the regulation of angio-miRNAs and subsequently blocking the HIF1-α/VEGF/MMP9 signaling pathway. The published evidence suggested the inhibitory effect of melatonin on hypoxia-induced cancer cell invasion and migration. Further studies are required to clarify the potential of melatonin in inhibiting the invasion and its underlying mechanisms.

#### 3.2.4. Other Effects of Melatonin on Hypoxia-Mediated Tumor Progression

Hypoxia changes the metabolic activity of cancer cells toward lower oxygen demand and elevated glucose uptake and lactic acid fermentation [54]. HIF-1 plays a major role in this scenario by up-regulating glycolytic enzymes (e.g., LDHA), GLUT1, GLUT3, and VEGF [55]. Moreover, the expression of PDK-1 and PDK-3, regulators of aerobic glycolysis, are increased by HIF-1 leading to proliferation and chemo-resistance of tumor cells. Conversely, melatonin as a regulator of redox homeostasis reduces ROS levels and consequently down-regulates HIF-1 and glycolysis-related enzymes such as GLUT1 and PFKFB3 [83,100]. In this regard, it was shown that melatonin treatment limits the expression of GLUT1 in breast cancer cells [101]. Sanchez et al. [102] also demonstrated that melatonin inhibited the Warburg effect in Ewing sarcoma cells by decreasing the glucose uptake and LDH activity. The inhibitory effect of melatonin on Warburg-type metabolism was also reported by Reiter and co-workers [103]. Another mechanism by which melatonin may influence glucose uptake into cancer cells is competition with glucose in binding to GLUT1 [104].

Hypoxia also stimulates levels of free intracellular Ca^2+^ and calmodulin (CaM) activity as well as the Ca^2+^/CaM signaling pathway [105]. Melatonin probably exhibits oncostatic actions by regulating Ca^2+^ signaling pathways via interacting with GPCR or modulating voltage-gated Ca^2+^ channels and also binding to CaM, tubulin, and retinoic acid receptors [67,106]. Moreover, melatonin regulates the Ca^2+^ signaling pathway via its ROS-scavenging activity [107].

## 4. Conclusions

It is well documented that hypoxia is involved in tumor progression via various mechanisms, including the induction of cancer cell invasion and migration, tumor angiogenesis, and modification of cell metabolism. On the contrary, melatonin can act as an anti-tumor agent partly through the inhibition of hypoxia-induced pathways. Herein, we discussed the possible signaling pathways by which melatonin inhibits hypoxia-induced cancer cell survival, invasion, migration, metabolism as well as tumor angiogenesis. The accumulated data overwhelmingly supported the idea that melatonin is an anti-cancer agent, independently or in combination with other chemotherapeutic agents. Considering melatonin efficacy and safety, it should be considered as part of the therapeutic regimen to treat certain types of cancer. Additional studies would further clarify the mechanisms by which melatonin acts as an oncostatic agent including the details of the proposed outline in this report. Plastic, 

## Data Availability

Not applicable.

## Abbreviations

HIF	hypoxia-inducible factor
GLUT	glucose transporters
ROS	reactive oxygen species
IGF	insulin-like growth factor
Cav-1	caveolin-1
Ang-2	angiopoietin 2
PDGF	platelet-derived growth factor
bFGF	basic fibroblast growth factor
ECM	extracellular matrix
MMPs	matrix metalloproteinases
COX	cyclooxygenase
LDHA	lactate dehydrogenase A
PDK	pyruvate dehydrogenase kinase
AC	adenylyl cyclase
PKA	protein kinase A
TGFα	transforming growth factor α
EDN1	endothelin 1
ANGPT	angiopoietin
ROCK1	Rho kinase 1
FAK	focal adhesion kinase
CaM	calmodulin
